# Sequencing Reveals Population Structure and Selection Signatures for Reproductive Traits in Yunnan Semi-Fine Wool Sheep (*Ovis aries*)

**DOI:** 10.3389/fgene.2022.812753

**Published:** 2022-03-07

**Authors:** Yi Guo, Jiachong Liang, Chunrong Lv, Yi Wang, Guoquan Wu, Xiangdong Ding, Guobo Quan

**Affiliations:** ^1^ Key Laboratory of Animal Genetics and Breeding of the Ministry of Agriculture and Rural Affairs, National Engineering Laboratory for Animal Breeding, College of Animal Science and Technology, China Agricultural University, Beijing, China; ^2^ Yunnan Provincial Genebank of Livestock and Poultry Genetic Resources, Yunnan Provincial Engineering Laboratory of Animal Genetic Resource Conservation and Germplasm Enhancement, Yunnan Animal Science and Veterinary Institute, Kunming, China

**Keywords:** Yunnan semi-fine wool sheep, sequencing, selection signatures, population structure, reproduction

## Abstract

Yunnan semi-fine wool sheep are among the most important cultivated sheep breeds in China. However, their population structure, genetic characteristics and traits of interest are poorly studied. In this study, we systematically studied the population characteristics and selection signatures of 40 Yunnan semi-fine wool sheep using SNPs obtained from whole-genome resequencing data. A total of 1393 Gb of clean data were acquired. The mapping rate against the reference genome was 91.23% on average (86.01%–92.26%), and the average sequence depth was 9.51X. After filtering, 28,593,198 SNPs and 4,725,259 indels with high quality were obtained. The heterozygosity rate, inbreeding coefficient and effective population size of the sheep were calculated to preliminarily explore their genetic characteristics. The average heterozygosity rate was 0.264, the average inbreeding coefficient was 0.0099, and the effective population size estimated from the heterozygote excess (HE) was 242.9. Based on the Tajima’s D and integrated haplotype score (iHS) approaches, 562 windows and 11,356 core SNPs showed selection signatures in the Yunnan semi-fine wool sheep population. After genome annotation and gene enrichment analysis, we found traces of early domestication in sensory organs, behavioural activity and the nervous system as well as adaptive changes in reproductive and wool traits under selection in this population. Some selected genes related to litter size, including *FSHR*, *BMPR1B* and *OXT*, were identified as being under selection. Specific missense mutations of the *FSHR* gene that differed from the reference genome were also identified in the population, and we found some SNP variations that may affect litter size. Our findings provide a theoretical basis for the conservation and utilization of Yunnan semi-fine wool sheep. Furthermore, our results reveal some changes common to sheep after domestication and provide a new opportunity to investigate the genetic variation influencing fecundity within a population evolving under artificial selection.

## Introduction

Yunnan semi-fine wool sheep are among the most important cultivated breeds in Yunnan Province, China. This breed has excellent wool quality, high adaptability and strong robustness. Currently, it is widely used to produce wool and meat products (Wang, 2009). From 1954 to 1971, to improve the wool fineness of ZhaoTong sheep, they were successively crossbred with Rambouillet hybrid sheep, Caucasian sheep, XinJiang fine-wool sheep, and New Zealand Romney sheep, among others. In 1977, Lincoln sheep were introduced to improve wool length, and in 1979, the ideal cross combination was obtained (National Commission On Livestock and Poultry Genetic Resources, 2011). In 2000, this breed was approved by the Chinese Livestock and Poultry Breed Approval Committee, becoming the first approved breed of coarse semi-fine wool sheep in China. Due to the long-term cultivation and growth of these sheep in alpine regions, they have developed high adaptability to cold climates, high altitudes and hypoxic conditions. In general, Yunnan semi-fine wool sheep are medium in body size, and their wool quality is comparable to that of Romney and Lincoln sheep. Due to its competitive advantages and rapid adaptation to the local environment, the population of Yunnan semi-fine wool sheep has been continuously expanded, with the population size exceeding 100,000 individuals in 2005 (Yuan and Sun, 2014). However, its conception rate and lamb survival rate are not high, and more than 90% of ewes give birth to one lamb per parity. Therefore, the improvement of reproductive traits is important for Yunnan semi-fine wool sheep.

Selection signatures are the imprints left on the genome of a species under long-term natural and artificial selection during the process of evolution. Genome selection signatures are an effective tool for studying the adaptability of domestic animals in different natural environments and exploring the genetic mechanisms underlying phenotypic differences. Various methods are currently used to assess genetic diversity and detect selection signatures. These methods can be divided into three categories depending on the different types of examined genomic information: methods based on the allele frequency spectrum, e.g., Tajima’s D (Tajima, 1989) and the composite likelihood ratio (CLR) (Nielsen et al., 2005); methods based on linkage disequilibrium (LD), e.g., extended haplotype homozygosity (EHH) (Sabeti et al., 2002) and the integrated haplotype score (iHS) (Voight et al., 2006); and methods based on population differentiation, e.g., the fixation index (FST) (Weir and Cockerham, 1984). In this study, we employed the iHS and Tajima’s D tests to detect selection signatures within the population.

With the development of sequencing technologies and the improvement of biological analysis methods, selection signature detection has been used to mine the imprints left by domestication and artificial selection and the genes associated with certain important traits (Porto-Neto et al., 2014; Liu et al., 2016; E et al., 2019; Abied et al., 2020; Zhao et al., 2020; Zhong et al., 2020; Zhu et al., 2020). However, due to certain limitations, it is difficult to obtain a complete picture of the germplasm and selection characteristics of indigenous sheep breeds in China. Therefore, the objectives of this study were to explore the population characteristics and genetic structure of Yunnan semi-fine wool sheep, to screen selection regions related to important traits and to identify variants by using genome-wide selection signature detection. Furthermore, the present study will provide a theoretical basis for the breeding and conservation of Yunnan semi-fine wool sheep.

## Materials and Methods

### Ethics Statement

The ethical committee of the Yunnan Animal Science and Veterinary Institute (Kunming city, Yunnan Province, China) approved all experiments in this study (201909006). In addition, during the study, all authors strictly complied with the Regulations on the Administration of Laboratory Animals (Order No. 2 of the State Science and Technology Commission of the People’s Republic of China, 1988) and the Regulations on the Administration of Experimental Animals of Yunnan Province (the Standing Committee of Yunnan Provincial People’s Congress 2007.10). There was no use of human participants, data or tissues.

### Animals and Phenotypes

A total of 40 female Yunnan semi-fine wool sheep with similar body weights (approximately 50 kg) and ages (3 years old) were sampled from the Laishishan sheep farm in Qiaojia City, Yunnan Province (N26°54′55.29″, E102°55′40.02″), which is one representative Yunnan semi-fine wool sheep farm in Yunnan Province, and genetic exchange (rams or sperm) was carried out with other Yunnan semi-fine wool sheep farms. The samples were unrelated according to the pedigree. Based on their litter sizes in two successive parities, these ewes were classified into two groups: ewes with a litter size of 1 (20 individuals) and those with a litter size of 2 (20 individuals). In addition, the sequencing data of 4 Zhaotong sheep and 3 Romney sheep were downloaded from the National Center for Biotechnology Information (NCBI) Sequence Read Archive (SRA) database to further explore the population structure of Yunnan semi-fine wool sheep.

### Sample Collection, DNA Extraction, and Whole-Genome Sequencing

Ear tissues of the selected sheep were collected, transferred to sterile centrifuge tubes, and stored in a freezer at −80°C. DNA was extracted from the sample of each individual and analysed following standard experimental procedures to guarantee quality. Sequencing libraries of each individual were constructed by Beijing BerryGenomics Biotechnology Co., Ltd., using the Illumina NovaSeq6000 PE150™ platform.

### Genomic Data Processing and SNP Calling

Trimmomatic software (version 0.38) (Bolger et al., 2014) was applied to remove adaptors and low-quality reads. After quality control, the reads of each sample were mapped to the GCF_002742125.1_Oar_rambouillet_v1.0 genome with the ‘mem’ algorithm of BWA software (version 0.7.17) (Li and Durbin, 2009). The bam and sorted bam files were generated using SAMtools (version 1.9) (Li et al., 2009). Then, the Genome Analysis Toolkit (GATK, version 3.7.0) (McKenna et al., 2010) pipeline was used to call SNPs and indels.

To ensure high quality of the variations for the following analysis, BCFtools (version 1.10.2) (Danecek and McCarthy, 2017) was used to filter the minimum or maximum number of alleles (bcftools view -m 2 -M 2) from autosomes and chromosome X. In addition, SNPs with minor allele frequencies lower than 0.01 or missing rates greater than 0.2 were excluded by VCFtools-0.1.16 (Danecek et al., 2011). To identify the functions of the variants, SnpEff 4.3 software (Cingolani et al., 2012) was utilized to annotate the filtered SNPs.

### Population Structure Analysis

Based on the filtered SNPs of Yunnan semi-fine wool sheep and their parental sheep breeds, principal component analysis (PCA), admixture analysis and LD decay analysis were performed to explore the population structure of Yunnan semi-fine wool sheep. For PCA, PLINK (v1.90b4) software (Purcell et al., 2007) was used to generate the input file (.bed.bim.fam). gcta64 software (v1.26.0) (Yang et al., 2011) was applied to construct a genetic relationship matrix (–make-grm) and calculate principal components. For admixture analysis, ADMIXTURE (version 1.3.0) (Alexander and Lange, 2011) was employed to carry out unsupervised hierarchical clustering. PopLDdecay software (version 3.41) was used to calculate LD decay, LD was evaluated on the basis of the squared coefficient of correlation (r^2^) between loci (Zhang et al., 2019), and the parameters were set as follows: -MaxDist 300 -OutType 1. All of the results were presented in graphs created using R software.

### Heterozygosity Rate, Inbreeding Coefficients and Effective Population Size

As important population parameters, the heterozygosity rate, inbreeding coefficient and effective population size were calculated in this study. PLINK (v1.90b4) software (Purcell et al., 2007) was used to estimate the heterozygosity rate, which was calculated with the formula $\frac{N\left(NM\right)-O\left(HOM\right)}{N\left(NM\right)}$ , where O(HOM) ,  E(HOM) and N(NM) represent the observed number of homozygotes, expected number of homozygotes and number of nonmissing genotypes, respectively, which were obtained by using PLINK. The inbreeding coefficients (*F*_*ROH*_) of individuals were estimated based on runs of homozygosity (ROHs). PLINK software was used to detect ROHs with the following parameters: 1) minimum density of 1 SNP per 100 kb, 2) minimum ROH length of 1000 kb, 3) 1 heterozygote allowed per window, 4) 5 missing calls allowed per window, 5) minimum number of SNPs in a window of 50, and 6) 0.05 missing calls allowed per window. The following formula was used to calculate *F*_*ROH*_:

$$F_{ROH}=\frac{\sum_{i}ROH_{i}}{L_{auto}}\;\;\;\;\left(McQuillan\;et\;al.,2008\right)$$

where $\underset{i}{\sum }ROH_{i}\;$ is the total ROH length on autosomes and $L_{auto}\;$ is the total length of autosomes. The effective population size (Ne) can indicate dynamic changes in population size, and Ne was estimated according to the random mating model based on the heterozygote excess (HE) method using the default parameters in NeEstimator (version 2.1) software (Do et al., 2014). NeEstimator calculates Ne as follows:

$$Ne=\frac{1}{2D}+\frac{1}{2\left(D+1\right)}\;\;\;\;\left(Pudovkin\;et\;al.,1996\right)$$

where Ne is the effective population size and D is Selander’s index, calculated as follows: $D_{j}\left(i\right)=\frac{H_{j}^{obs}\left(i\right)-H_{j}^{\exp}\left(i\right)}{H_{j}^{\exp}\left(i\right)}$ . $H_{j}^{\exp}\left(i\right)$ is the expected heterozygote frequency of allele i at locus j. The actual D value is the weighted mean value, calculated as follows: $D=\frac{\sum w_{j}D_{j}}{\sum_{j=1}^{k}\left(n_{j}-1\right)\sqrt{N_{j}}}$ , where w_j is the weight of locus j and is calculated as $w_{j}=\sqrt{N_{j}}\frac{n_{j}-1}{n_{j}},\;$ where $\;N_{j}$ is the sample size, and $n_{j}$ is the number of alleles at locus j.

### Detection of Selection Signatures

Selection signature detection within populations can reveal selection dynamics and the history of evolution. In this study, two metrics, Tajima’s D (Tajima, 1989) and the iHS (Voight et al., 2006), were utilized to identify selection signatures in the whole genome of Yunnan semi-fine wool sheep. Tajima’s D is based on neutral mutation theory. When Tajima’s D is equal to 0, it indicates that the population is in a neutral evolutionary state, whereas Tajima’s D values lower or greater than 0 indicate that the population has experienced purifying selection events/population expansion or balancing selection, respectively. Tajima’s D values were calculated in nonoverlapping 50-kb sliding windows. Only the windows with the 1% highest and the 1% lowest Tajima’s D values were identified as subject to selection. Different from the single locus-based Tajima’s D metric, the iHS is mainly based on haplotypes. A negative iHS value indicates that the mutated allele may be affected by positive selection. In this study, haplotypes were first constructed by using SHAPEIT (Delaneau and Marchini, 2014), and iHS statistics were then calculated using Selscan software (version 1.2.0) (Szpiech et al., 2014). Thereafter, the iHS values were standardized to follow a standard normal distribution, and the statistics exceeding the 0.1% quantile ( |Z(iHS) value|=3 ) indicated that the core SNP experienced selection.

### Functional Annotation of Selected Regions

Based on the core SNPs or windows under selection detected based on the iHS and Tajima’s D analyses, selected regions were defined for the bioinformatics analysis. From each core SNP detected based on iHS analysis, the region was extended 20 kb upstream and downstream to define the selected region. Under the Tajima’s D approach, each selected 50-kb sliding window was extended 150 kb upstream and downstream to define the selected region. Gene information in the selected regions was obtained using the Biomart database (http://asia.ensembl.org/biomart/martview/). Kyoto Encyclopedia of Genes and Genomes (KEGG) pathway and Gene Ontology (GO) enrichment analyses were performed for further gene function analysis using DAVID Bioinformatics Resources 6.8 (Huang et al., 2009) (https://david.ncifcrf.gov/). The GO terms and KEGG pathways with P values less than 0.05 were considered significant.

### Mutation Analysis of Selected Genes Relevant to Reproductive Traits

In this study, 40 Yunnan semi-fine wool sheep were classified into two groups on the basis of litter size: 1 lamb (20 individuals) or 2 lambs (20 individuals) in two successive parities. To investigate whether SNP mutations or frequency differences existed between the two groups, three significantly selected genes (*FSHR*, *BMPR1B*, and *OXT*) related to reproductive traits were studied. PLINK (v1.90b4) software was employed to perform a standard case (individuals with two lambs)/control (individuals with one lamb) association analysis using Fisher’s exact test with the option plink --file gene --fisher.

## Results

### Genetic Variants in Yunnan Semi-Fine Wool Sheep

In the present study, a total of 40 female Yunnan semi-fine wool sheep were sequenced. After filtering out contaminated reads, including adaptors, low-quality reads and reads with an N ratio greater than 10%, 1393 Gb of clean data were acquired, and 35,178,304 raw SNPs and 5,256,453 raw indels were obtained. The mapping rate against the reference genome was 91.23% on average (86.01%–92.26%), and the average sequence depth was 9.51X (8.09X∼12.10X) (see Supplementary Material S1 for details). After filtering, 28,593,198 SNPs and 4,725,259 indels with high quality were retained.

**FIGURE 1:**
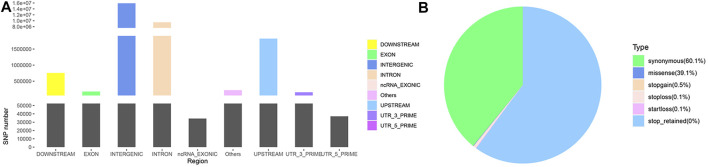
SNP annotation of Yunnan semi-fine wool sheep. **(A)** Genomic annotation of SNPs according to SnpEff. “Others” contained a few other “splice site acceptor,” “splice site donor” and “splice site region” sites. **(B)** Pie chart of SNPs annotated in exonic regions.

Following annotation, 27,623,037 SNPs on autosomes and 970,161 SNPs on chromosome X (Supplementary Material S2) were obtained. These SNPs were partitioned according to their locations: intergenic (15,897,566; 56.03%), intronic (9,491,317; 33.45%), exonic (177516; 0.63%) and other gene regulatory regions (Figure 1A). Among the SNPs in exonic regions, as shown in Figure 1B, synonymous mutations constituted the overwhelming majority (60.07%), followed by missense mutations (39.14%), and the other types of observed mutations included stop-gain, stop-loss, and start-loss mutations (less than 1%). The transition/transversion (Ts/Tv) ratio was 2.4721.

### Genetic Diversity of the Yunnan Semi-Fine Wool Sheep Population

To describe the genetic diversity of the Yunnan semi-fine wool sheep population, two metrics were used. For the whole population, the average heterozygosity rate and inbreeding coefficient were 0.264 (0.231–0.374) and 0.0099 (0.0004–0.0239), respectively (see Supplementary Material S4 for details), indicating that the heterozygosity rates and inbreeding coefficients varied greatly within the population. The effective population size estimated from the HE of the Yunnan semi-fine wool sheep population using the lowest allele frequency of 0.05 as the threshold was 242.9 (95% CI: 232.6–254.2).

### Population Structure Analysis

**FIGURE 2:**
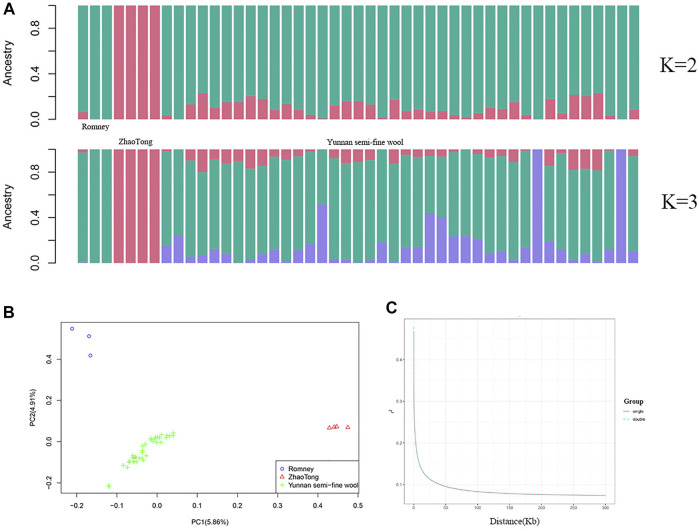
Genetic differentiation of 40 Yunnan semi-fine wool sheep. **(A)** ADMIXTURE analysis of Yunnan semi-fine wool sheep and their parental sheep breeds (Romney and Zhaotong sheep). **(B)** PCA of 40 Yunnan semi-fine wool sheep and their parental sheep breeds using 710,412 common SNPs located on autosomes. **(C)** LD decay curves (based on r^2^) of two groups (single or double lambs).

The population structure is illustrated in Figure 2. ADMIXTURE analysis indicated that the ancestors of Yunnan semi-fine wool sheep included Romney and Zhaotong sheep, with Romney sheep contributing more (K = 2); Figure 2A also demonstrates other ancestors of Yunnan semi-fine wool sheep (K = 3). Moreover, PCA (Figure 2B) further showed that the population of Yunnan semi-fine wool sheep was aggregated and separated from Romney and Zhaotong sheep, indicating that it is a new breed. LD decay analysis of two groups with litter sizes of 1 or 2 lambs (Figure 2C) confirmed the absence of obvious population stratification within Yunnan semi-fine wool sheep, and the difference between litter sizes might be due to ongoing selection.

### Detection of Genome-Wide Signatures of Selection

**FIGURE 3:**
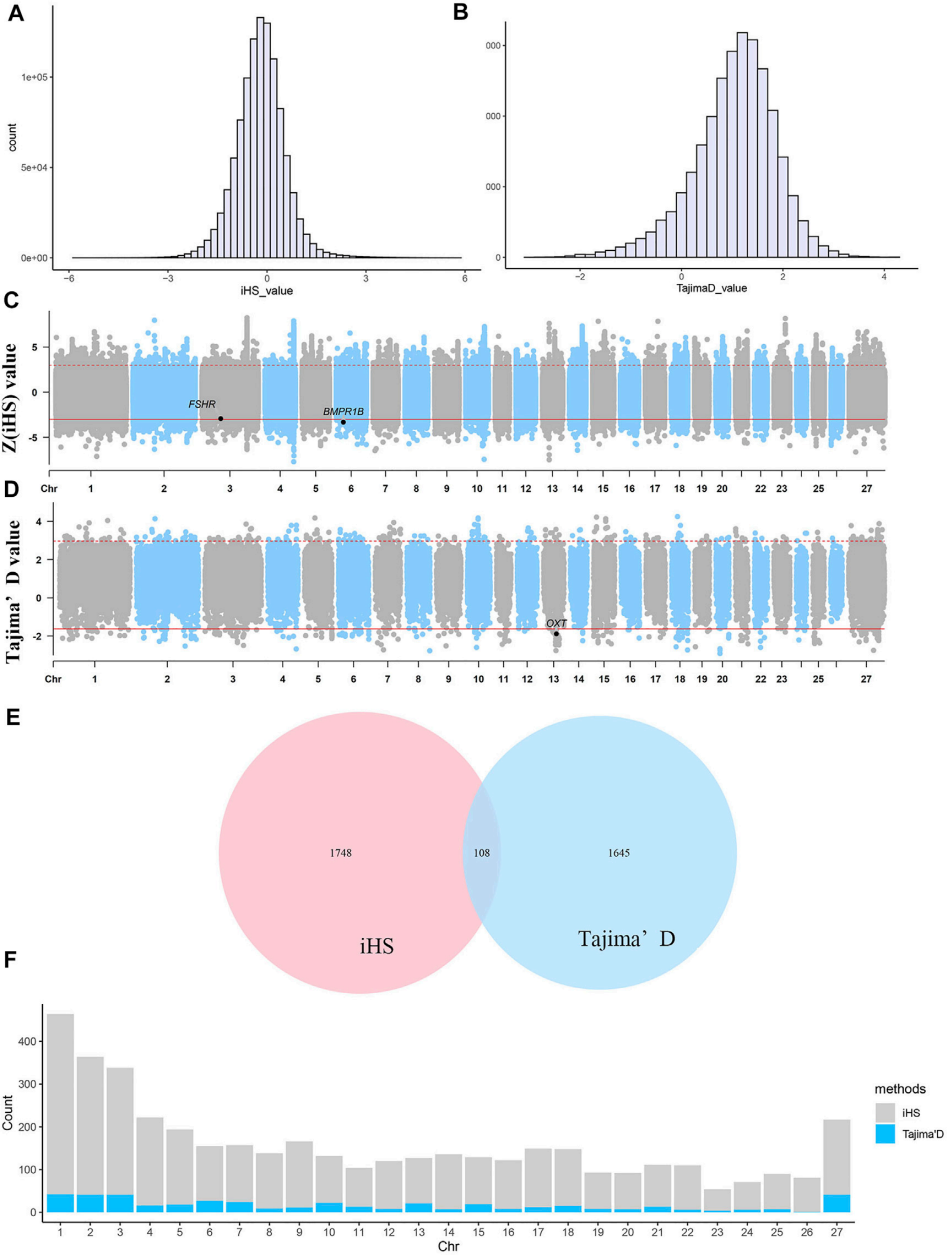
Selective sweep analysis of 40 Yunnan semi-fine wool sheep. **(A)** Distribution of iHS values for selected regions. **(B)** Distribution of Tajima’s D values for selected regions. **(C)** Manhattan plot of Z(iHS)  values of sheep. The dashed line denotes a threshold of |Z(iHS) value| = 3 (*p* = 0.001). **(D)** Manhattan plot of Tajima’s D values. The black line is the “suggestive line”: the top 1% of values are >2.97345, and the bottom 1% of values are < −1.63182. **(E)** The 108 common selected genes detected by both the iHS and Tajima’s D approaches. **(F)** The distribution of the selected SNPs on each chromosome obtained by the iHS and Tajima’s D approaches. In the figure, “27” on the X-axis represents chromosome X.

As shown in Figure 3, regardless of whether the iHS or Tajima’s D approach was used, the observed selection signatures were distributed across all chromosomes. Under the iHS approach, the 0.1% quantile ( |Z(iHS) value|=3 ) was used as the threshold to extract selected core SNPs. A total of 11,356 core SNPs showing the strongest selection were identified, accounting for 1% of all selected SNPs. The iHS values ranged from −7.69 to 8.26 (Figures 3A,C). Considering the LD among SNPs, the selected SNPs within each 50-kb interval were combined into one selected region. In total, 3512 selected regions were identified. Chromosomes 1, 2, 3 and 4 harboured the most selected regions, i.e., 422, 323, 297 and 206 selected regions, respectively (Figure 3F).

A total of 56,115 nonoverlapping 50-kb windows were assessed, and only the top and bottom 1% of the windows with high Tajima’s D values were determined to be regions of selective sweeps (Figures 3B,D). A total of 562 windows showing the strongest selection and 449 equivalent selected regions were identified by combining the selected windows. Selected regions were identified on all chromosomes (Figure 3F). Among all chromosomes, chromosomes 1, 2, 3 and X harboured the most selected windows, i.e., 42, 41, 41 and 41 windows, respectively. The most significant regions were located on chromosome 20 from 16.70–16.85 Mb, with an average Tajima’s D value of −2.77432, and this region harboured 495 SNPs. Five known genes were identified within this putative selective sweep region, namely, *CUL7*, *KLC4*, *MEA1*, *MRPL2* and *PTK7*, which may regulate cell activity and microtubule motor activity.

### Functional Enrichment of Candidate Genes

According to the two methods of selection signature detection, 1856 genes were found in the 20-kb regions upstream and downstream of the core SNPs according to the iHS test, and 1753 selected genes were obtained in the 350-kb intervals upstream and downstream of the selected windows based on the Tajima’s D test. We combined the genes obtained via the two methods described above, and a total of 108 genes were identified (Figure 3E). To evaluate the functions of the selected genes obtained by the two methods, gene annotation and enrichment analyses (GO and KEGG) were conducted.

**FIGURE 4:**
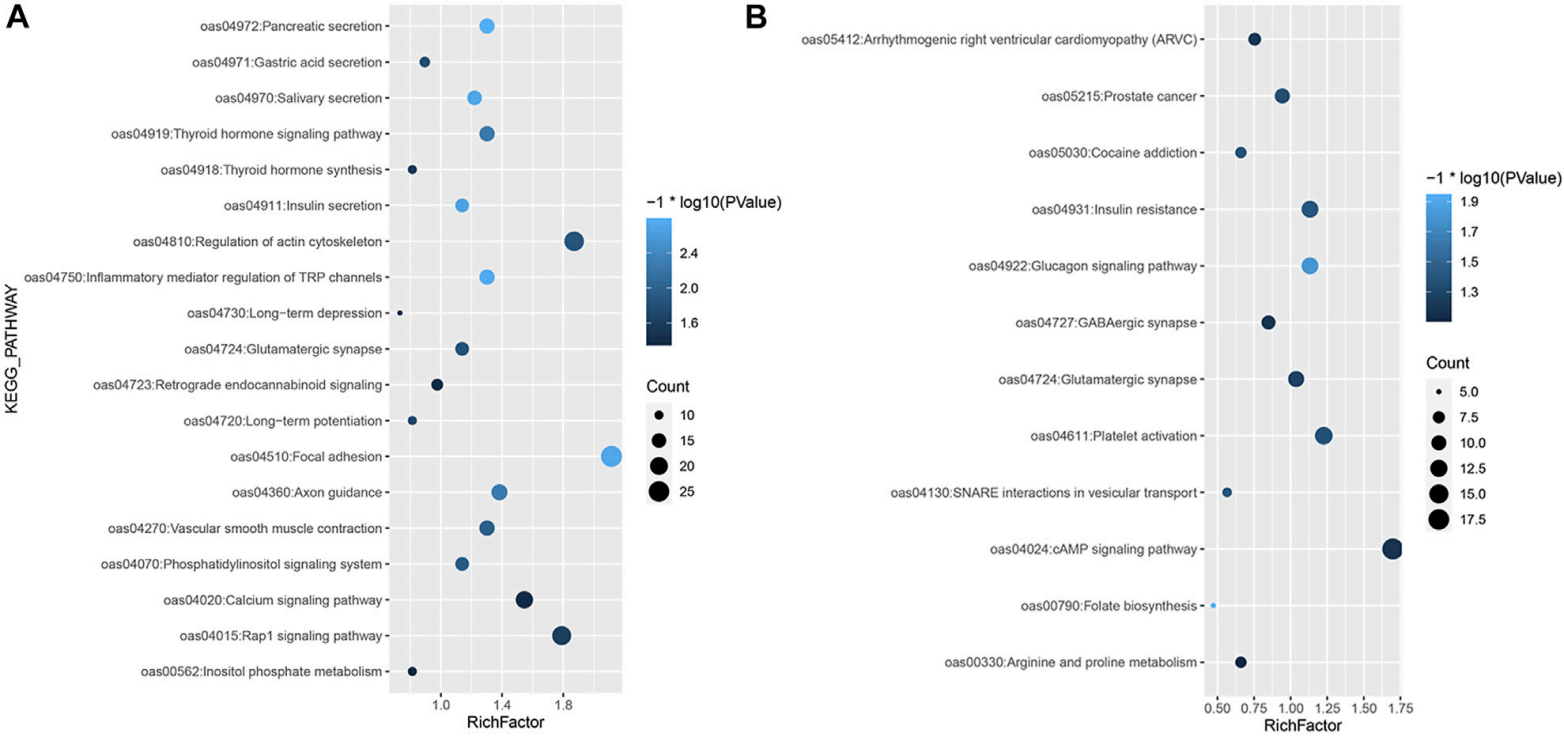
KEGG enrichment analysis of selected genes. **(A)** KEGG analysis of the selected genes based on the **(A)** iHS and **(B)** Tajima’s D approaches.

For the iHS, the enrichment analysis identified a total of 53 significant GO terms (Supplementary Material S3; Figure 1), namely, 13 cellular component terms, 17 molecular function terms, and 23 biological process terms. Notably, most of the selected genes were enriched in the categories of immune system processes, intracellular signal transduction and neural synapses. In the KEGG analysis results, 19 pathways were annotated, as shown in Figure 4A. Some known reproduction-related, domestication-related and wool-related pathways were found to be significantly enriched, including the vascular smooth muscle contraction, calcium signalling, salivary secretion, gastric acid secretion, long-term depression and long-term potentiation pathways.

Among the results based on Tajima’s D method, 18 significant GO terms were identified (Supplementary Material S3; Figure 2), including olfactory receptor activity, immune process and dendrite morphogenesis terms. A total of 12 KEGG pathways were obtained (Figure 4B), including folate biosynthesis related to reproductive traits and the GABAergic synapse pathway related to inhibition of the nervous system.

Overall, our results revealed traces of early domestication in Yunnan semi-fine wool sheep, such as modifications in sensory organs, behavioural activity and the nervous system, as well as adaptive changes in economic traits, including reproductive and wool traits, under artificial selection (Table 1).

**TABLE 1:** GO terms or KEGG pathways associated with domestication, reproduction and immune system processes.

		Category	Term	Count	P Value
Domestication	iHS	GOTERM_BP_DIRECT	Synapse organization	5	0.0499
		KEGG_PATHWAY	Salivary secretion	15	0.0020
		KEGG_PATHWAY	Glutamatergic synapse	14	0.0148
		KEGG_PATHWAY	Gastric acid secretion	11	0.0170
		KEGG_PATHWAY	Long-term potentiation	10	0.0197
		KEGG_PATHWAY	Long-term depression	9	0.0329
		KEGG_PATHWAY	Retrograde endocannabinoid signalling	12	0.0418
	Tajima’s D	GOTERM_MF_DIRECT	Olfactory receptor activity	36	0.0130
		GOTERM_BP_DIRECT	Positive regulation of dendrite morphogenesis	3	0.0446
		KEGG_PATHWAY	Glutamatergic synapse	11	0.0526
		KEGG_PATHWAY	GABAergic synapse	9	0.0650
Reproduction	iHS	KEGG_PATHWAY	Vascular smooth muscle contraction	16	0.0107
	Tajima’s D	KEGG_PATHWAY	Folate biosynthesis	5	0.0112
Immune system process	iHS	KEGG_PATHWAY	Inflammatory mediator regulation of TRP channels	16	0.0018
		KEGG_PATHWAY	Thyroid hormone signalling pathway	16	0.0058
		KEGG_PATHWAY	Thyroid hormone synthesis	10	0.0306
Wool	iHS	GOTERM_BP_DIRECT	Negative regulation of BMP signalling pathway	7	0.0393
		GOTERM_BP_DIRECT	Positive regulation of peptidyl-tyrosine phosphorylation	9	0.0355
	Tajima’s D	GOTERM_BP_DIRECT	Hyaluronan catabolic process	3	0.0446
		GOTERM_BP_DIRECT	Regulation of protein phosphorylation	6	0.0041
		GOTERM_MF_DIRECT	Protein tyrosine phosphatase activity	10	0.0378

### Mutation and Association Analysis of Genes Related to Reproduction

Genes related to reproductive traits, such as follicle-stimulating hormone receptor (*FSHR*), bone morphogenetic protein receptor type 1B (*BMPR1B*) and oxytocin/neurophysin I prepropeptide (*OXT*), were identified as being under selection in this study. *OXT* was identified by the Tajima’s D test, and *BMPR1B* and *FSHR* were identified by the iHS test. Based on the whole-genome sequencing results obtained for Yunnan semi-fine wool sheep, the polymorphic loci in the exons of the *BMPR1B*, *FSHR* and *OXT* genes were analysed by comparison with the reference genome (Table 2).

**TABLE 2:** Mutations in the *BMPR1B* and *FSHR* genes in exonic regions of the Yunnan semi-fine wool sheep genome.

Gene	Chromosome	Position	cDNA variation	Position of mRNA	Amino acid variation	Mutation type	db SNP	MAF
*BMPR1B*	6	33998156	1059A > C	1059	Arg353Arg	Synonymous variant	rs429416173	0.43
		33992536	1416C > A	1416	Thr472Thr	Synonymous variant	rs413854373	0.28
		34009717	825C > T	825	Ser275Ser	Synonymous variant	rs598261578	0.04
		34009732	810T > C	810	Tyr270Tyr	Synonymous variant	rs159952533	0.41
		34011008	543A > G	543	Thr181Thr	Synonymous variant	rs427897187	0.33
		34011011	540G > A	540	Arg180Arg	Synonymous variant	rs408447622	0.08
		34051987	52G > A	52	Ala18Thr	Missense variant	rs605658565	0.16
*FSHR*	3	80789093	696C > T	696	Ser232Ser	Synonymous variant	rs412817989	0.41
		80794664	1113C > T	1113	Phe371Phe	Synonymous variant	rs416291965	0.11
		80605490	28G > A	28	Ala10Thr	Missense variant	rs399253678	0.25
		80789089	692G > A	692	Arg231His	Missense variant	rs399612350	0.40
		80789180	783T > G	783	Phe261Leu	Missense variant	rs422112895	0.04
		80789251	854C > T	854	Thr285Ile	Missense variant	rs398233545	0.06
*OXT*	13	—	—	—	—	—		

**FIGURE 5:**
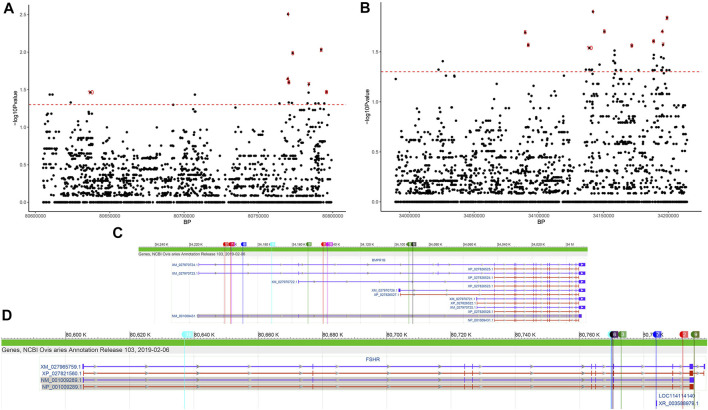
SNPs of the *FSHR* and *BMPR1B* genes differing between the single- and double-lambing groups identified using Fisher’s exact test. Manhattan plot of **(A)** *FSHR* and **(B)** *BMPR1B* SNPs differing between the single- and double-lambing groups. The red line indicates *p* = 0.05. The location of the top 10 significant loci on the **(C)** *FSHR* and **(D)** *BMPR1B* gene structures.

In the *FSHR* gene, a total of 2437 SNPs were identified. Six mutations were detected in its exonic region (Table 2), four of which were missense mutations (c.28G < A, c.692G > A, c.783T > G, and c.854C > T, known mutation loci). At the same time, we calculated the distribution of minor alleles in the two groups based on litter size. Twenty-three SNPs with significant differences as detected by Fisher’s exact test were finally obtained (Figure 5A). Although these SNPs were all located in intronic regions, they are probably related to reproductive performance in the two groups.

In the *BMPR1B* gene, among the 2645 identified SNPs, 7 SNP mutations were located in the exonic region, only one of which was a missense variant (c.52G > A, known mutation locus, rs605658565), and 44 SNPs showed significant differences between the single- and double-lambing groups (Figure 5B). In the *OXT* gene, there were only 3 identified SNPs (Chr13:54216488, 54216953, and 54216953). Although none of the SNPs showed significant differences, we found that in the two-lamb group, the frequency of minor alleles increased *de novo*.

## Discussion

### Population Situation of Yunnan Semi-Fine Wool Sheep

Yunnan semi-fine wool sheep were the first coarse-grade semi-fine wool sheep breed cultivated in China. In the last 2 decades, the breed has become an indispensable component of sheep germplasm resources in China. This study is the first to explore the population characteristics, genetic structure and selection signatures based on sequencing data of this breed. We used the heterozygosity rate and inbreeding coefficient to evaluate genetic diversity within the population. ROHs are continuous homozygous segments, and they can reflect the inbreeding level of a population at the genome level. Using ROHs to calculate the inbreeding coefficient (*F*_*ROH*_) is more accurate than estimating the inbreeding coefficient from pedigree data (Kim et al., 2015). In this study, the average inbreeding coefficient (0.0099: 0.0004–0.0239) was lower than that of other sheep breeds, e.g., Chaka sheep (*F*_*ROH*_ = 0.032) (Cheng et al., 2020), Italian Alpagota sheep (*F*_*ROH*_ = 0.053) (Mastrangelo et al., 2018)) and other species, e.g., white leghorn (*F*_*ROH*_ = 0.28) (Zhang et al., 2020) and Laiwu pigs (*F*_*ROH*_ = 0.133) (Fang et al., 2021), indicating that the population structure of Yunnan semi-fine wool sheep is ideal. Combining the average inbreeding coefficient and heterozygosity rate, we could preliminarily infer that although these two parameters varied greatly within the Yunnan semi-fine wool sheep population, sufficient genetic diversity was maintained overall during cultivation and artificial selection. This conclusion is well supported by the Ne results. In the long-term selection process, the greater the selection intensity is, the smaller the effective population size and the lower the genetic diversity of the population. According to previous studies, when the effective population size is below 50 individuals, the population is threatened (Meuwissen and Woolliams, 1994). According to our results, the Ne of Yunnan semi-fine wool sheep is large enough to indicate a low risk of excessive inbreeding at present.

The breeding of Yunnan semi-fine wool sheep is carried out under a strict system: new genetic materials cannot be introduced into the core group, and sheep in propagation groups must be obtained from foundation seed farms, which maintains the good breeding status of these sheep. However, the herd size in breeding farms is small, e.g., only 1010 and 270 sheep in the Lashishan breeding farm and Xiaohai breeding farm in Qiaojia County, respectively, both of which are representative breeding farms of Yunnan semi-fine wool sheep in Yunnan Province. Such a small population size will lead to inbreeding, resulting in a decrease in the effective population size.

### Genomic Signatures of Selection in Yunnan Semi-Fine Wool Sheep

Genetic variation profoundly affects phenotypic variation. When either natural or artificial selection occurs, it will leave traces in the genome. Therefore, we adopted intrapopulation detection methods (Tajima’s D and iHS) to discover genome-wide footprints caused by natural and artificial selection in Yunnan semi-fine wool sheep. In this study, overlap of the selected genes simultaneously identified by the two approaches was observed in a few cases (108 genes, Figure 3E). The iHS approach shows higher efficiency in detecting partial sweeps; thus, it can detect an event in which a favourable allele increases rapidly from a low frequency but has not yet reached a fixed state (Pritchard et al., 2010). The Tajima’s D test is especially powerful for the detection of fixation signatures. According to the characteristics of the above two signature detection methods, the two approaches can be employed together to identify more traces of selection in the genome. Considering the short breeding history of Yunnan semi-fine wool sheep, positive selection may still be affecting some genes related to wool, meat quality and reproductive traits. Furthermore, some alleles for genes associated with domestication and adaptation may be fixed.

Domestication refers to the process by which wild animals come to be maintained under domestic conditions, in which they reproduce over generations and are used by humans. Domestic animals undergo fundamental changes in their phenotypes, morphology, and behaviour relative to those of wild animals (Naval-Sanchez et al., 2018). Some key traits were selected and fixed in most domestic sheep breeds in the early stages of domestication. The changes in these traits mainly manifest as reductions in brain volume and weight, which lead to dulled sensory organ function, more docile personalities and slower activities, such as vision, smell and motor abilities (Kruska, 1996). In this study, we identified some biological pathways associated with nervous system regulation and olfactory receptor activity, consistent with the results of previous studies (Axelsson et al., 2013; Li et al., 2020).

Due to the spread of domestic sheep with human migration activities worldwide, some traits associated with specific human needs have been fixed, leading to greater diversity of some phenotypes. In the Yunnan semi-fine wool sheep population investigated in this study, in addition to the findings related to domestication, we also found many selected regions and novel functional genes that may be responsible for traits such as reproduction and wool production. Some biological pathways associated with reproductive traits, including vascular smooth muscle contraction and folate biosynthesis, were identified, which may be related to the development of the endometrial vascular system and pregnancy (Cullinan-Bove et al., 1993).

In previous studies on a segregated flock based on QTL analysis and GWAS mapping, some mutations such as *FecB* (Mulsant et al., 2001), *FecX* (Galloway et al., 2000), and *FecG* (Nicol et al., 2009) that may affect ovulation in sheep were identified. Interestingly, we also found that the *BMPR1B* gene was located in a selected region in this study. The *BMPR1B* gene encodes a member of the bone morphogenetic protein (*BMP*) receptor family of transmembrane serine/threonine kinases and regulates animal cell growth and differentiation, embryonic development and reproductive performance (Wilson et al., 2001). Since the mutant *FecB* [nonsynonymous substitution (Q249R)] form of the sheep *BMPR1B* gene was proven to be the main gene regulating high-fertility performance in Booroola sheep, researchers have attempted to identify the *FecB* gene in some indigenous breeds such small-tail Han and Hu sheep (Chu et al., 2011). However, some researchers have reported no significant link between this gene and high fertility traits in breeds such as Tan sheep (Chong et al., 2019). In this study, the *FecB* mutation was not detected in Yunnan semi-fine wool sheep, suggesting that this *BMPR1B* gene mutation may not affect the reproductive performance of the breed. Of course, it is also possible that the examined sample size was too small for detection of this mutation in this study. Nevertheless, we detected 44 SNPs with significantly different frequencies between the single- and double-lambing groups. However, these SNPs were mainly located in intronic regions. Many mutations in intronic regions have previously been found to (Figure 5D) lead to functional changes through aberrant splicing (Busslinger et al., 1981; Vaz-Drago et al., 2017), so it is necessary to expand the studied population or conduct experimental verification.

Two other genes that participate in reproduction-related hormone secretion are *FSHR* and *OXT*. The *OXT* gene encodes a precursor protein that is processed to produce oxytocin and neurophysin I. This precursor seems to be activated as it is transported along the axon to the posterior pituitary. This hormone causes the contraction of smooth muscle during parturition and lactation and functions as a neurotransmitter in the central nervous system, playing a role in cognition, tolerance, adaptation, and complex sexual and maternal behaviours (Gimpl and Fahrenholz, 2001). In the present study, there was little evidence that this gene was associated with litter size, but we did find differences in SNPs in this gene region between the two litter size groups, suggesting that *OXT* may be related to litter size. Most relevant studies have shown that the *FSHR* gene is closely related to reproductive traits, such as ovarian function (Laan et al., 2012) and testicular development (Lend et al., 2010), in mammals. SNPs in the 10th exon of *FSHR* are significantly correlated with fertility traits of the Chinese native pig breed Xiaomeishan (Wu and Wang, 2012). Mutations in the 5′ flanking region of the ovine *FSHR* gene may also be associated with litter size (Pan et al., 2014). In this study, we identified specific missense mutations in the Yunnan semi-fine wool sheep population that differed from the reference genome. However, their functions still need to be further explored. On this basis, 23 SNPs with significantly different frequencies were also identified between the single- and double-lambing groups. All of these SNPs were located in intronic regions (Figure 5C), but their effect on lambing size needs to be further investigated.

## Conclusion

In conclusion, using second-generation sequencing technology, the population structure of Yunnan semi-fine wool sheep was detected, and strategies for the conservation of this breed were proposed. In addition, some genes related to adaptation and reproductive traits were shown to have experienced strong selection. The results of this study increase our knowledge of the genetic basis of litter size in Yunnan semi-fine wool sheep, shed light on the changes in heritable phenotypes during the processes of adaptation and artificial breeding, and provide a theoretical basis for the breeding and conservation of Yunnan semi-fine wool sheep.

## Data Availability

The datasets presented in this study can be found in online repositories. The names of the repository/repositories and accession number(s) can be found below: NCBI, PRJNA783661.
