# Supplementary material for: Sequencing Reveals Population Structure and Selection Signatures for Reproductive Traits in Yunnan Semi-Fine Wool Sheep (Ovis aries)
Source: Front Genet. 2022 Mar 7;13:812753. doi: 10.3389/fgene.2022.812753 (PMC8957090; doi:10.3389/fgene.2022.812753)
Supplement: Supplementary file 4 [file DataSheet1.docx]

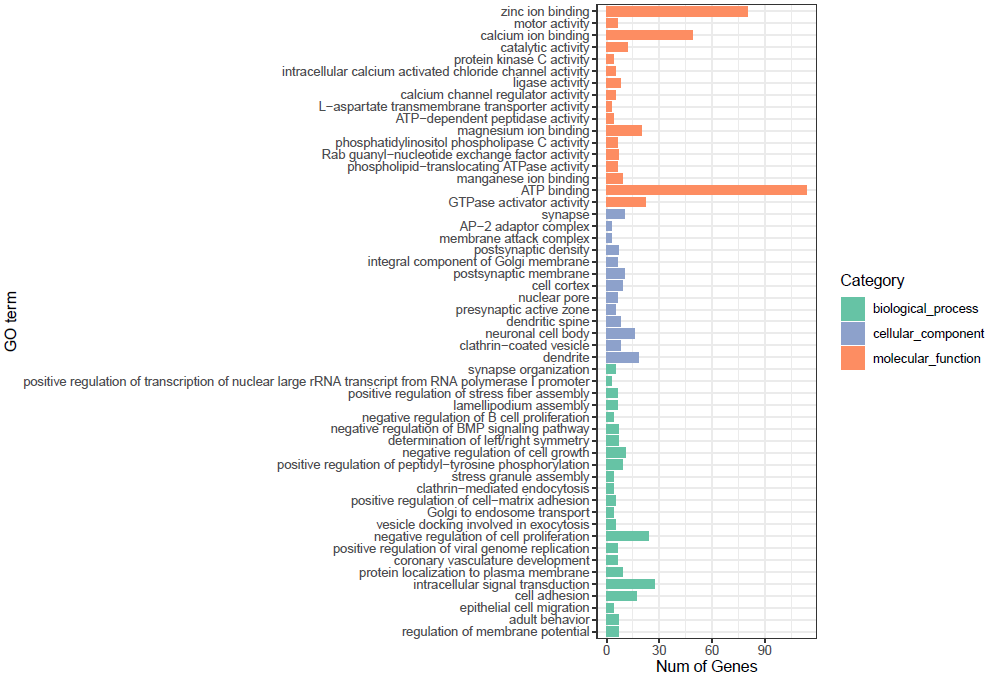


Figure 1 Gene Ontology analysis of selected genes obtained by iHS method


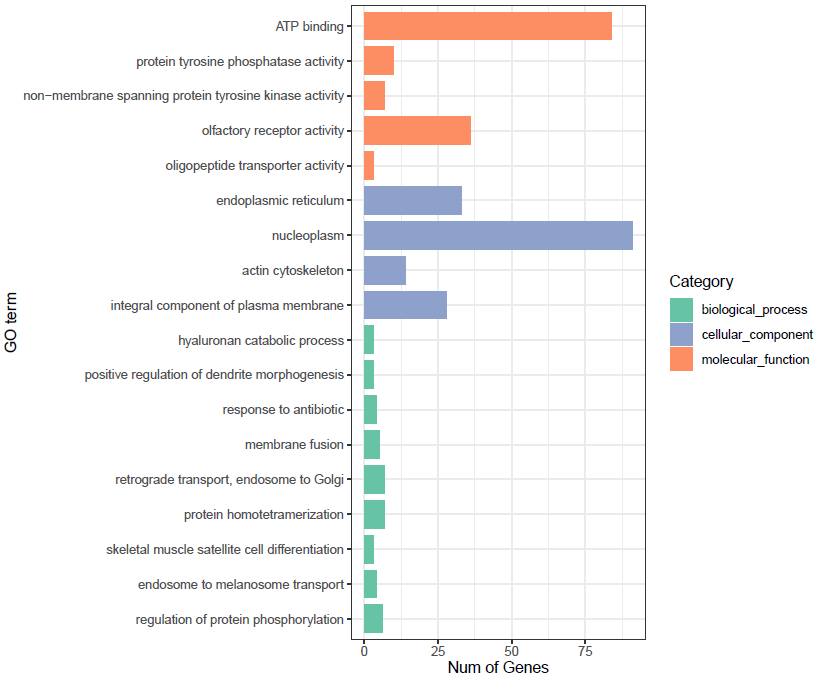


Figure 2 Gene Ontology analysis of selected genes obtained by Tajima’D method
